# Predictive simulation of sit-to-stand based on reflexive-controllers

**DOI:** 10.1371/journal.pone.0279300

**Published:** 2022-12-30

**Authors:** David Muñoz, Cristiano De Marchis, Leonardo Gizzi, Giacomo Severini

**Affiliations:** 1 School of Electrical and Electronic Engineering, University College Dublin, Dublin, Ireland; 2 Insight Centre for Data Analytics, Dublin, Ireland; 3 Department of Engineering, University of Messina, Messina, Italy; 4 Department of Engineering, Roma Tre University, Rome, Italy; 5 Institute for Modelling and Simulation of Biomechanical Systems, University of Stuttgart, Stuttgart, Germany; 6 Centre for Biomedical Engineering, University College Dublin, Dublin, Ireland; Tokai University School of Medicine, JAPAN

## Abstract

Sit-to-stand can be defined as a set of movements that allow humans to rise from a sitting position to a bipedal standing pose. These movements, often categorized as four distinct kinematic phases, must be coordinated for assuring personal autonomy and can be compromised by ageing or physical impairments. To solve this, rehabilitation techniques and assistive devices demand proper description of the principles that lead to the correct completion of this motor task. While the muscular dynamics of the sit-to-stand task have been analysed, the underlying neural activity remains unknown and largely inaccessible for conventional measurement systems. Predictive simulations can propose motor controllers whose plausibility is evaluated through the comparison between simulated and experimental kinematics. In the present work, we modelled an array of reflexes that originate muscle activations as a function of proprioceptive and vestibular feedback. This feedback encodes torso position, displacement velocity and acceleration of a modelled human body with 7 segments, 9 degrees of freedom, and 50 actuators. We implemented two controllers: a four-phases controller where the reflex gains and composition vary depending on the kinematic phase, and a simpler two-phases controller, where three of the kinematic phases share the same reflex gains. Gains were optimized using Covariance Matrix Adaptation. The results of the simulations reveal, for both controllers, human-like sit-to-stand movement, with joint angles and muscular activity comparable to experimental data. The results obtained with the simplified two-phases controller indicate that a simple set of reflexes could be sufficient to drive this motor task.

## Introduction

Sit-to-stand (STS) is a motor task where a set of neural commands drive the muscles of the lower limbs and the torso to generate torques that lead the human body from a seated position to an upright stance. Being STS one of the key activities of daily living [1], weakness in the lower limbs and the torso, affecting control or strength and thus the execution of the STS task, severely hinder the autonomy of individuals. A wide range of impairments can trouble STS, with sarcopenia (loss of muscular mass by ageing) being considered one of the most critical, affecting the 8.8% and 17.5% of the elderly women and men, respectively [2]. Enhancing our understanding of the biomechanical characteristics of STS and how this task is controlled could lead to the development of better assistive devices or more efficient therapies which can help impaired or elderly adults to achieve the successful execution of this task.

With such intention, in 1990, Schenkman et al. studied the STS movement in healthy individuals [3]. They quantitively described the kinetics and kinematics of the task and separated them in four phases, *flexion momentum* (generation of momentum by leaning forward the torso), *momentum transfer* (use momentum to rise the trunk), *extension* (extension of hips and knees), and *stabilization* (finalization of the movement towards a stable posture).

In 2016, Caruthers et al. described the muscle dynamics that originate these kinematic phases [4]. They explored the muscle-induced acceleration through experimental EMG and 3D modelling. As they described, gluteus maximus, biceps femoris, and adductor magnus are the major contributors for the *momentum transfer* phase, while plantar flexors are required for lifting the body in the *extension* phase.

Even though the relationship between kinematics, kinetics and muscular activations during STS is known [4], the underlying high-level control strategies and low-level neural pathways used to execute the movements remain undetermined. Current proposals about the nature of the neurophysiological control of STS include contributions from the sensorimotor network [5], or the interconnectivity between spinal central pattern generators (CPGs) and reflexes [6]. Moreover, successful STS requires the integration of several sensorial inputs with psychological processes [7].

*Predictive simulations* can help understanding the contribution of different neural sources in the control strategies for STS. Applied to neuromuscular models, allow for testing different hypotheses on control strategies and neural controllers, and to determine an optimal model by maximizing posterior probability [8]. In the case of STS, a musculoskeletal model like the one developed in [4] can be used to propose different neural control architectures and assess if the emerging simulated kinematics and muscular activations match experimental data.

In the present work, we developed predictive simulations for the STS based on a reflex controller. Pure sensorial feedback control applied to predictive simulations has been an efficient method to replicate human mechanics since Geyer & Herr published their gait muscle-reflex model [9]. Here we tested whether series of multiple sensory neurons each connected to a single muscle could explain human STS, as a mean to unravel reflex contribution to the control of task.

While Geyer & Herr used muscle force as main sensorial feedback, we describe the STS reflex controller as a set of vestibular and proprioceptive reflexes. In humans, the sensorial information from semicircular canals and otolith organs is integrated with neck proprioception to provide a body centred reference information [10]. As the musculoskeletal model we used is neckless (see Musculoskeletal model and simulation scenario section), this information is represented as the translation, velocity, and acceleration of the centre of mass (CoM) of the torso segment, while muscle length reflexes regulate excessive muscle stretching.

We developed two reflex-based *neural controllers* for predictive simulations of STS. The first model is built as a strict representation of the kinematic phases identified in literature (4-phases controller), with reflex composition and gains that are specific for each phase, while the second groups the possible reflex-muscle interactions in two phases, by collapsing the *momentum transfer*, *extension*, and *stabilization* phases together (2-phases controller). The first phase, *flexion momentum*, is too kinematically demanding to be included in this merged phase. The simulated STS movements obtained through these models aim at shedding additional light on whether: 1) a reflex-based dynamic can replicate the kinematics and muscular activations of the STS, and 2) the kinematic phases necessarily reflect an underlying neurophysiological organization, or they emerged from a simpler organization. The simulations were designed with the aim of achieving STS and keeping balance after finishing the task to test the stability of the execution. The last phase of each controller would be responsible of this *standing task*, and simulations unable to perform it were discarded.

The controllers were evaluated by applying them to a musculoskeletal model developed in OpenSim [11]. The simulations were run using the customizable framework SCONE [12], where the sets of gains and offsets were optimized using Covariance Matrix Adaptation Evolutionary Strategy (CMA-ES) [13], following the constrains dictated by a composite cost function underlying the task goals. We validated our controllers by comparing the kinematics and muscular activations relative to the simulated STS movements and those recorded from healthy individuals.

## Methods

### Experimental data

A sample of 13 healthy subjects (2 F, 36.9 ± 10 years, 177.2 ± 6.6 cm, 79 ± 11.7 kg) was enrolled for an experimental sit-to-stand protocol. The recruitment did not aim at balancing the numbers for gender and for age representation, given that no changes in results between genders and different age groups were expected for our protocol [14]. The experiment consisted of executing twice the 30 seconds sit-to-stand protocol [15]. The experiments were executed at the laboratory of bioengineering Biolab3 of the University Roma Tre. All subjects gave written informed consent for participation in the study. The whole experimental procedure was conducted according to the principles of the Declaration of Helsinki, and it was approved by the ethics committee of the University Roma Tre.

**Fig 1:**
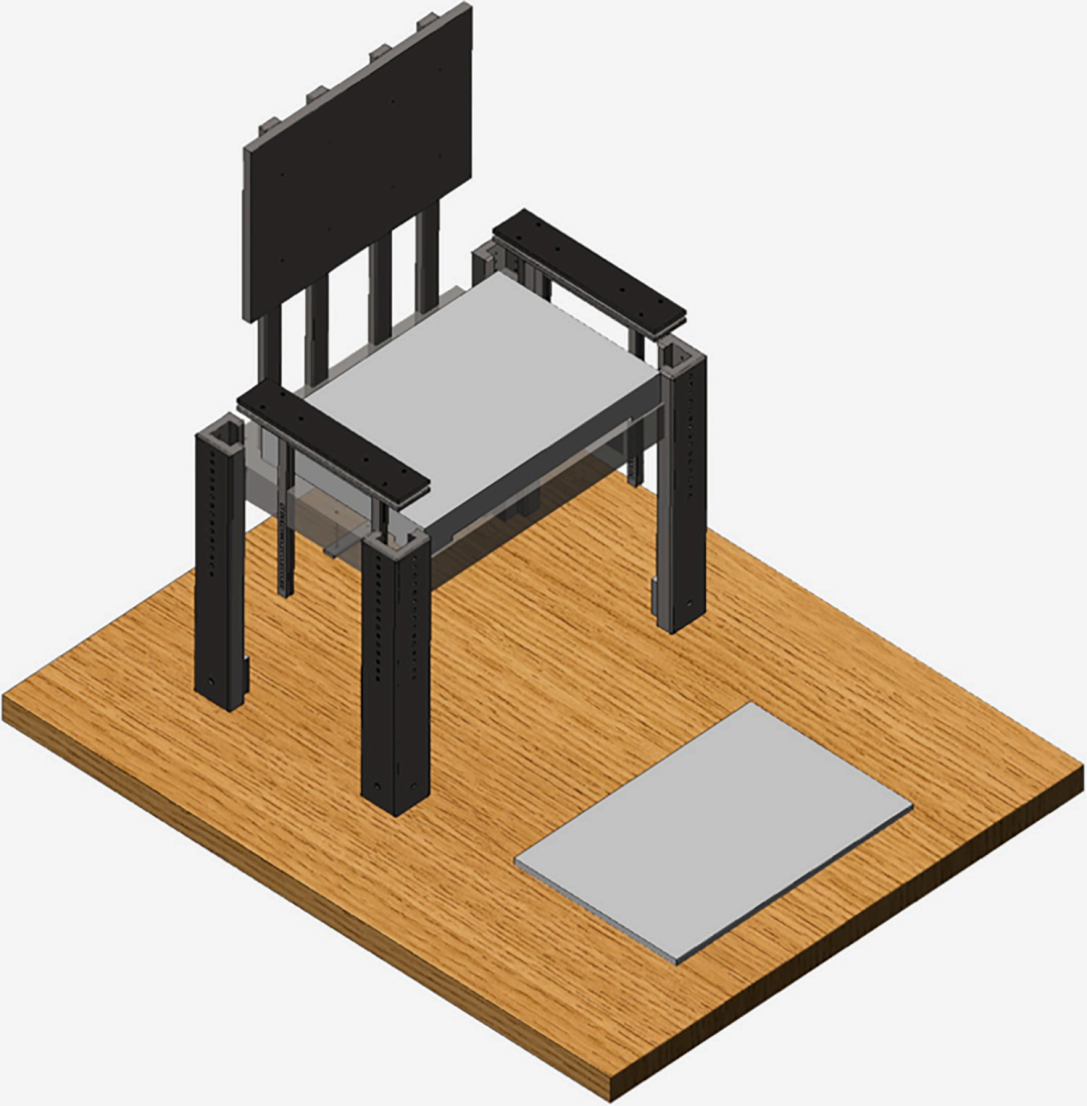
A 3-dimensional render of the BENCH apparatus.

The experiment started with the subject comfortably sitting with the arms folded across their chest. At the “go” signal, each subject was asked to seamlessly stand up and sit down with a self-selected yet sustained pace for a duration of 30s. The subjects were instructed to perform the sit-to-stand movements in a clean way, reaching a full stand position before starting the upcoming stand-to-sit phase. The protocol was administered on the **BENCH apparatus** (schematic representation in Fig 1), an instrumented chair equipped with two force plates (BTS P6000), one on the seat and one on the ground. The height of the chair is adjustable and was set to match the height of a standard office chair (45 cm). Reaction forces from the ground and seat force plates were recorded with a sampling frequency of 250Hz and digitized at 16 bits. The body of the chair is composed by a set of custom-designed PA6 Nylon elements can be interfaced with EMG sensors, a motion capture system (see below) and, optionally, accelerometers (not used in this experiment).

Kinematic data were recorded with a BTS SMART DX6000 system (BTS Bioengineering, Milan, Italy) equipped with 8 infrared cameras. A set of 15 reflective markers were placed over the sacrum and bilaterally over the shoulder, anterior iliac spinae, great trochanter, lateral femoral condyle, head of the fibula, lateral malleolus and fifth metatarsal head, to calculate the joint kinematics in the sagittal plane (i.e., trunk bending, hip flexion/extension, knee flexion/extension and ankle plantar/dorsal flexion). Kinematics were recorded with a sampling frequency of 250 Hz. Synchronized EMG data were recorded at a sampling frequency of 1 kHz with a wireless BTS FREEEMG 1000 system from the following 8 muscles of the right lower limb: Soleus (SOL), Gastrocnemius Medialis (GAS), Peroneus Longus (PER), Tibialis Anterior (TA), Vastus Lateralis (VL), Rectus Femoris (RF), Biceps Femoris (BF) and Gluteus Maximus (GLU). Bipolar recordings were performed by means of Ag/AgCl electrodes (Kendall ARBO) placed according to international recommendations [16] with an inter-electrode distance of 2cm.

### Musculoskeletal model and simulation scenario

The musculoskeletal model that has been used in the simulations was adapted from the OpenSim [11] models *Human0914* and *Gait2392* [17]. The model is constituted by a 9 DOFs, seven-segmented human body, with a height of 1.8 m and a weight of 80 kg. The DOFs are 6 pin joints for hips, knees, and ankles, representing the rotation of each joint in the sagittal plane. The additional 3 DoFs are the three free movements of the musculoskeletal model respect to the ground in the sagittal plane (anteroposterior translation, longitudinal translation, and pitch rotation).

**Fig 2:**
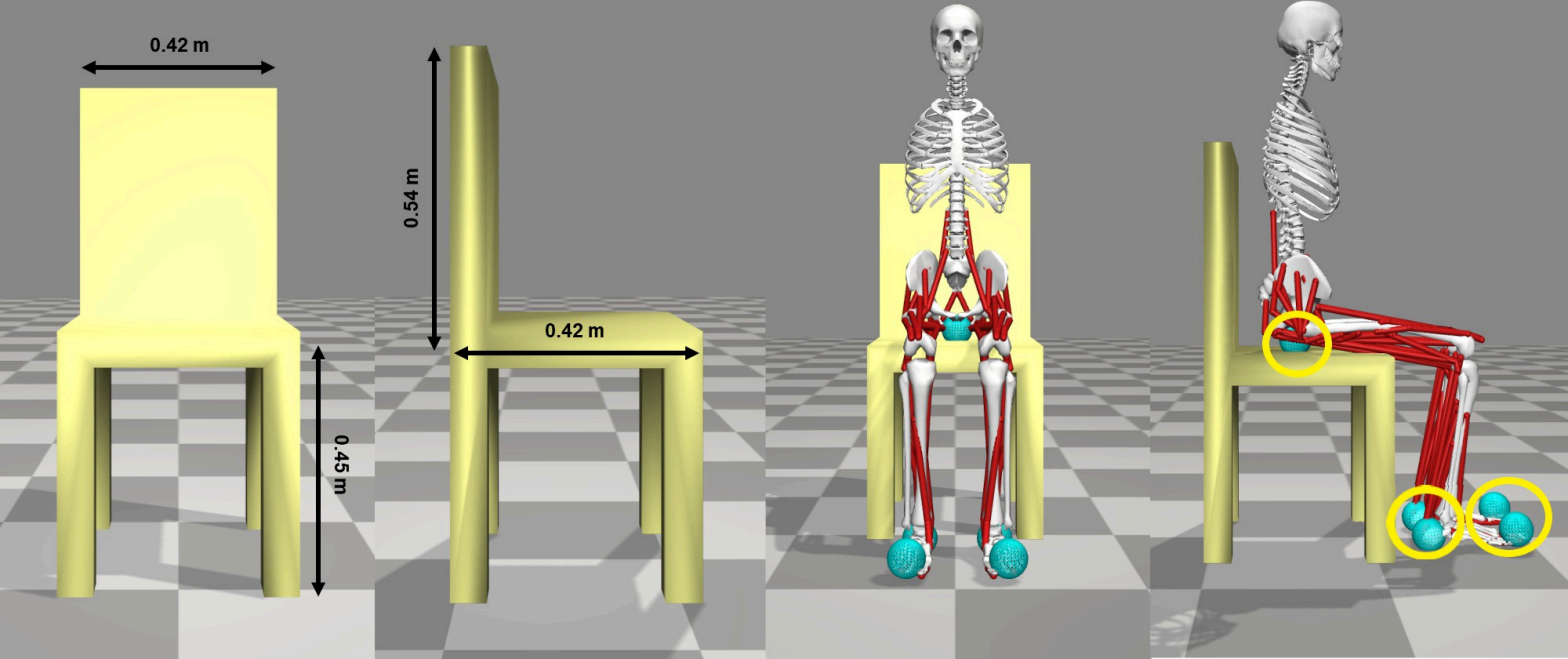
Simulation scenario. Measurements of the chair, musculoskeletal model and contact geometry (yellow circles).

For each joint, the zero is set to the corresponding angular position of the subjects when stay in quite stance at the end of the STS movement. The model includes feet, shanks, thighs, and a single segment representing torso, pelvis, trunk, and head. The arms are not included. The model is actuated by 50 Hill-type muscle-tendon units representing 38 muscles (19 each side). They are *gluteus medius*, *gluteus maximus*, *biceps femoris long head*, *biceps femoris short head*, *adductor magnus*, *iliacus*, *psoas*, *rectus femoris*, *vastus intermedius*, *vastus lateralis*, *medial gastrocnemius*, *lateral gastrocnemius*, *soleus*, *tibialis anterior*, *flexor digitorum longus*, *flexor hallucis longus*, *erector spinae*, *semimembranosus*, and *semitendinosus*. Their modelling parameters are specified in [17]. The other elements of the simulation scenario are the *chair* and the *ground*. The chair is 0.45 m in height from the ground to the sit, 0.54 m in length from the front to the back and has a width of 0.42 m and it is linked to the ground by a weld joint. The contact geometry is formed by a *ground platform*, *a chair platform*, and five *contact spheres*. Contact spheres are 4 cm of radius and are placed 4 cm forwards, 2 cm upwards, and 1.4 cm onwards from the centre of the toes, 1 cm forwards, 2 cm upwards, and 0.5 cm outwards from the centre of the calcaneus, and 7.5 cm backwards and 12 cm downwards from the centre of the pelvis. The feet contact spheres react with the ground platform. The pelvis contact sphere reacts with the chair platform and was placed 3.5 cm above the chair, while feet contact spheres were placed 4 cm above the ground. The musculoskeletal model, the chair, and the contact geometry are presented in Fig 2.

The simulations start with the model in a static sitting position imitating the average observed experimental initial posture. The initial angles of the joints are set to 12°, 85°, and 61° for hip, knee, and ankle, respectively. Angles are defined so to increase as the joint extends according to [18]. In the experimental subjects, the initial leaning of the torso is due to the angle of the hip and the lumbar flexion, but the musculoskeletal model does not have a DOF for lumbar flexion. For this reason, we set the initial hip angle so the angular position of the torso with respect to the positive *x* axis matches the average torso inclination of the subjects. The initial knee and ankle angles in the model were set to the average observed from the experimental data.

### Sit-to-stand phases

The study of the kinematics of STS has been often paired with the characterization of the dynamic events that drive the different parts of the task. Schenkman et al. [3] organized STS as a task that starts when the individual breaks the sitting position by moving forward, and finishes when the upright standing position is achieved, and all the movements associated with rising conclude. They divided this cycle in four consecutive phases. In the phase I, *flexion momentum*, momentum is created by leaning the torso forward and ends when the buttocks leave the seat (*lift-off*). Phase II, *momentum transfer*, uses this momentum to move the torso upwards and forwards to the new base of support (BoS), the feet. This phase ends when ankle reaches maximum dorsiflexion. In Phase III, *extension* of hips, and knees achieves the vertical pose, and finishes when the hips stop extending (0°/s). During the last phase (IV), *stabilization*, the residual motion is dispelled.

The four-phases structure is convenient not just for analysing the STS, but also for developing models. Here we propose a control scheme where the phases, as expressed in [3], represent subtasks that need to be achieved, either through phase-dependent reflexes (4-phases controller) or through a simpler set of reflex-based control rules (2-phases controller). However, in this control framework, the transitions between these phases cannot be fully implemented as proposed [3]. The phases coded for both controllers represent the same subtask goals but are bounded differently. We refer as the kinematic phases those proposed by the previous literature [3], and as the modelled phases the ones we implemented in the models (Table 1).

**Table 1:** Subtask and end of the kinematic and the modelled phases.

Phase	Subtask	Kinematic description	4-phases controller	2-phases controller
**Flexion momentum**	Create momentum by leaning the torso forwards	Buttocks lift off	*Θ*_*hip*_ > 77°	*Θ*_*hip*_ > 77°
**Momentum transfer**	Use the momentum to move the body forwards and upwards and place it above the new BoS (feet)	Maximum dorsiflexion	*p*_*torso*,*ml*_ > = *p*_*feet*,ml_* (m)	End of simulation (merged phase)
			*v*_*torso*,*linear*,*ml*_ < = 0 m/s	
			*Θ*_*shank*_ < 90°	
**Extension**	Extend the body a normal stance posture	Hips stop extending	*Θ*_*knee*_< = 5°	
			*Θ*_*hip*_ < = 0°	
**Stabilization**	Dispel all the forces related to rising	Movements related with rising disappear	End of simulation	
**Standing**	Keep upright position until the end of the simulation	No part of the STS cycle		

*Mediolateral.

**Fig 3:**
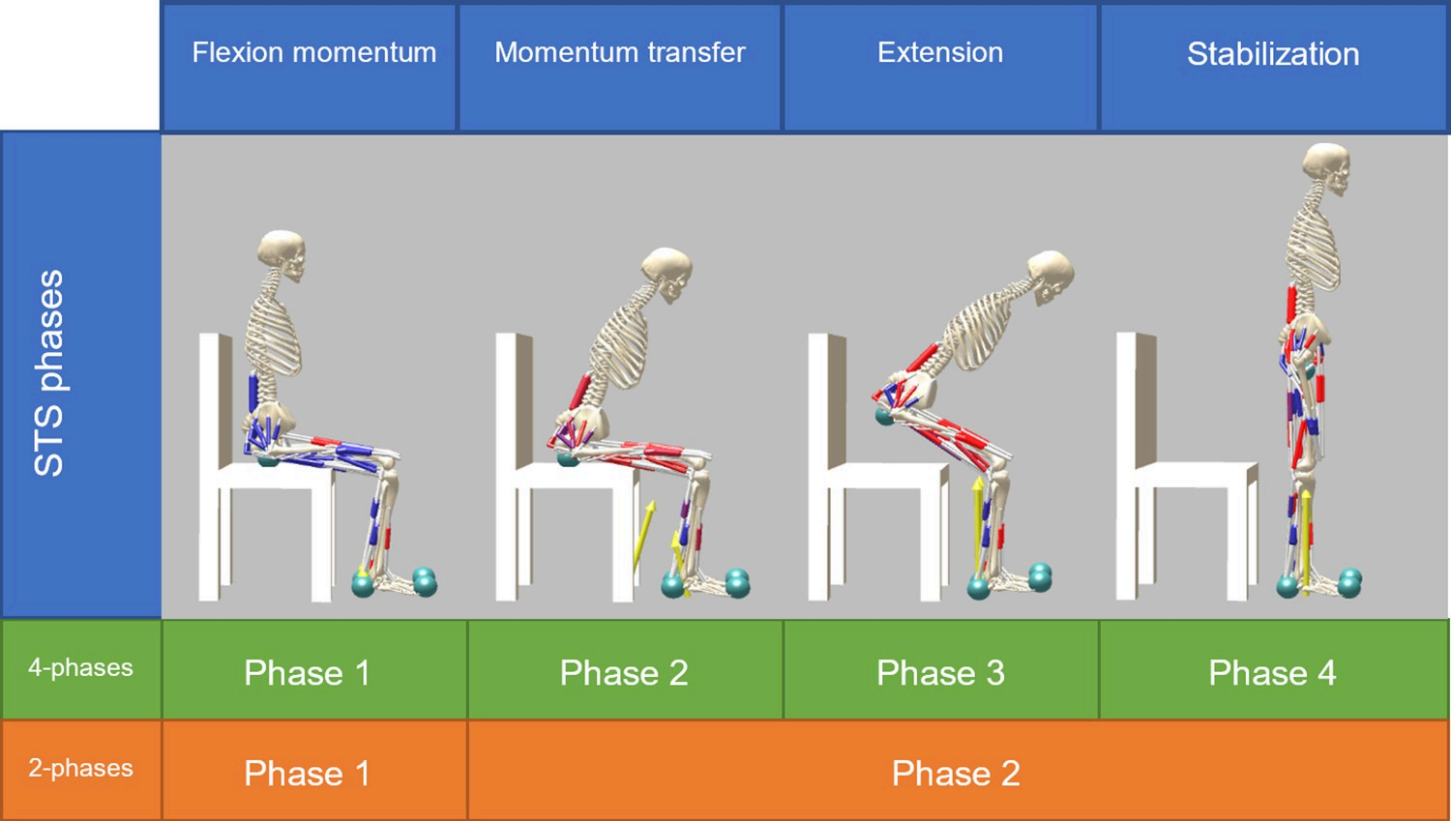
STS cycle described according to their kinematics and to the controllers.

At the end of the *flexion momentum* phase a neural controller requires a control variable that indicates that the torso has enough momentum for initiating *momentum transfer*. To signal that, we consider whether the torso achieved an acceleration of 0.8 ms^-1^ by the moment the hips reach an angle of 77°. The maximum dorsiflexion of the ankle cannot be used as the end of the *momentum transfer* phase as this value cannot be predicted with the current setup. We used the instant when the CoM of the torso segment arrives to the new BoS (i.e., when the position of the CoM is placed forward the calcaneus for first time) as an alternative event for the transition to the *extension* phase. Additionally, we included other events to ease the transition to the *extension* phase as the backwards torso speed and the forwards leaning of the shank. The end of the third phase can be implemented similarly to the kinematic description, taking the instant when the knee extension falls below 5°. We added, as another condition for the transition to the *stabilization* phase, a check on whether the hips are extended more than 0°. The *stabilization* phase lasts until the end of the simulation, fixed at a maximum of 20s. The 2-phases controller presents a simpler organization with respect to the kinematics phases or the 4-phases controller (Fig 3). While the *flexion momentum* phase and the transition to the next phase is identical to the 4-phases controller, the other three phases are merged. In this case, the *merged* phase is responsible of the kinematics for the rest of the STS cycle.

We used the kinematic phases in the data analysis, as they are the consequence of the execution of the controllers (see Data analysis section). For the kinematic phases, the standing task that happens after the end of the STS is supposed to be an additional phase, but for the models, this task is part of the *stabilization* or the *merged* phase.

### Reflex controller

The controllers are designed to translate vestibular and muscle length reflexes into inputs for the hill-type muscles. These reflexes are modelled as synapses between the sensorial inputs and the efferent α-motoneurons of a specific muscle, following the general linear form:

$$A_{m}=A_{0,m}+G_{m}F_{m}$$ (1)

where *A*_*m*_ is the stimulation for the *m* muscle, *A*_*0*,*m*_ is the tonic stimulation for that muscle, *F*_*m*_ is a particular sensorial feedback signal, and *G*_*m*_ is its related gain.

According to Angelacki & Cullen [10], otolith and semicircular canals codify linear and angular acceleration of a head-centred reference frame. After sensorial processing and integration, these sensorial inputs transform into acceleration, velocity, and position information of the head. In a second step of integration with proprioceptive information coming from the neck muscles and joints, these inputs can represent the acceleration, velocity, and position of the torso.

The reflex controller uses this integrated body-centred modality to calculate the activation for each actuator as a function of the position, velocity, and acceleration of the model torso in both longitudinal and mediolateral directions.

We hypothesized that the gains for each sensorial input and the offset values are phase dependent, thus each phase of the 4-phases and 2-phases controller has its own set of gains and its own version of the following equation (based on Eq 1):

$$A_{m}=A_{0,m,n}+(k_{p,i,m,n}\;k_{v,i,m,n}\;k_{a,i,m,n})(\begin{array}{c} p_{i}\\ v_{i}\\ a_{i} \end{array})+k_{l,m}(L_{m}-L_{0,m})$$ (2)

where *A*_*m*_ is the stimulation for the actuator *m*, *A*_*0*,*m*_ is the tonic stimulation for that actuator, *k*_*p*,*i*,*m*,*n*_, *k*_*v*,*I*,*m*,*n*_, and *k*_*a*,*i*,*m*,*n*_ are the gains for position (*p*_*i*_), velocity (*v*_*i*_), and acceleration (*a*_*i*_) signals, respectively, in the i-th direction, longitudinal or mediolateral, of the segment of the torso, and in the n-th phase of the controller. Other reflex control is muscle length *L*_*m*_ with an offset reference *L*_*0*,*m*_, and a gain *k*_*l*,*m*_. Gains and offset values (*L*_*0*,*m*_, and *A*_*m*,_) are to be optimized. The controllers are symmetrical, the same activation *A*_*m*_ is used for both actuators in each side. The parameter *n* changes according to the conditions described in Table 1 in a control sequence. The sequence is described for both controllers as:

$$n=\left\{ \begin{array}{cc} 2 & if\;\theta_{hip}>77{}^{\circ},\\ 3 & if\;p_{torso,ml}\geq p_{feet,ml}\&v_{toso,linear,ml}\leq 0{}^{\circ}\&\theta_{shank}<90{}^{\circ},\\ 4 & if\;\theta_{knee}\leq 5{}^{\circ}\&\theta_{hip}\leq 0{}^{\circ},\\ 1 & otherwise \end{array}\right.$$ (3)

$$n=\left\{ \begin{array}{l} 2\;if\;\theta_{hip}>77{}^{\circ},\\ 1\;otherwise \end{array}\right.$$ (4)

Each case of Eqs 3 and 4 supposes a non-returning point, where the phases are reached consecutively and only in one direction.

Each muscle is associated with its specific reflexes, characterized by specific gains. Reflexes are active for all muscles in all the phases with the exclusion of the *Flexion momentum* phase. In this phase the *rectus femoris* was selected as the main contributor to produce the forward traction, because the musculoskeletal model does not include the rectus abdominis. The reflexes linked to the other hip flexors, the iliacus and psoas, had to be set to zero during the *Flexion Momentum* because their inclusion caused the optimization to never converge. In the rest of the phases (or phase, for the 2-phases controller) all the muscles are activated.

Eq 2 is expressed differently along the four phases, depending on which feedback is used by the controller during each of them (see Table 2). During the *flexion momentum* phase of both controllers, the angular position, velocity, and acceleration of the torso with respect to the ground are used in the feedback loop to generate the appropriate momentum. For the *momentum transfer* and the *extension* phases of the 4-phases controller, the reflexes are based on the translational position of the torso on the longitudinal and the sagittal axis. For the *stabilization* phase, we use the sagittal translation from the pelvis and torso to drive the control of the posture. Each phase has a different set of vestibular gains, even those that has the same structure. However, all the phases share the same muscle length feedback gains that counteracts overactivity produced by the other inputs. For the 2-phases controller, the control variables are the same as the 4-phases controller for the *flexion momentum* phase, while the *merged* phase uses the same control variables than the *momentum transfer* phase of the 4-phases controller.

**Table 2:** Control variables for both controllers.

4-phases controller		2-phases controller	
Phase	Control variables	Phase	Control variables
**Flexion momentum**	Torso angular position, velocity, and acceleration, x axis	**Flexion momentum**	Torso angular position, velocity, and acceleration
**Momentum transfer**	Torso linear position, velocity, and acceleration, x and y-axis, x axis	**Merged phase**	Torso linear position, velocity, and acceleration, x and y-axis
**Extension**	Torso linear position, velocity and acceleration, x and y-axis		
**Stabilization**	Torso and pelvis, linear position, velocity, and acceleration, x axis		

### Cost function

The cost function was implemented to maximize sagittal and longitudinal translation while keeping balance. The elements of the cost function are also phase-dependent, with the elements for each phase designed to optimize the achievement of the specific subtask objective.

For the 4-phases controller, during *Flexion momentum*, forward suboptimal acceleration is penalized (< 1.2 ms^-2^). During *momentum transfer*, torso and pelvis backwards movement are penalized together with hip (< -11°) and knee (< 0°) extension, and falling, while in the *extension* phase hip (< -11°) extension and falling are penalized. During *Stabilization*, bad postures (i.e., torso leaning forwards while the pelvis is place behind the feet) and falling are penalized. In all of them, we put constraints to feet movement and hopping.

In the 2 phases controller, the cost function is implemented following the same principles shown in the 4-phases controller. The penalizations for the first phase do not change, and forward suboptimal acceleration is penalized (< 0.8 ms^-2^). For the *merged* phase the implementation by subtasks is not possible, thus phases are represented as conditional statements in the cost function. This implementation allows for the different control variables and penalizations to be phase-dependent, even though the controller remains in the same phase and the set of gains and offset values do not change. To avoid falling backwards immediately after the lift-off, torso and pelvis backwards movements are penalized only when the hips are flexed beyond 44°. When the hips are flexed in a range between 8° and 77°, the model is identified with the kinematic *extension* phase, and knee and hip flexion velocity cannot fall below 10°/s and 0°/s, respectively. Once the hips have been extended (< = 8°), its angle are forced to remain in a range of 8° of extension/flexion and knees are not allowed to flex more than 11°. Conditions aside, torso falling, and feet movement are penalized in the 4-phases controller.

Note that we did not use an energy expenditure minimization criterion in the cost function for either controller. The phase-dependent elements of the cost functions for the two controllers are presented in Tables 3 and 4.

**Table 3:** Phase-dependent penalizations in the cost function for the 4-phases controller optimization.

Phase	Penalizations	Cost function
**Flexion momentum**	Suboptimal acceleration	*a*_*torso*,*linear*,*ml*_^a^ < = 1.2 m/s^2^
	Feet movement	*v*_*toes*,*linear*,*long*_^b^ > 0.2 m/s
**Momentum transfer**	Pelvis backwards movement	*v*_*pelvis*,*linear*,*ml*_ < = 0 m/s
	Feet movement, hopping	*v*_*talus*,*linear*,*long*_ > 0.1 m/s
		*v*_*toes*,*linear*,*ml*_ > 0.15 m/s
	Hip and knee extension	*Θ*_*hip*_ < -11°
		*Θ*_*knee*_ < 0°
	Torso falling	*p*_*torso*,*long*_ < *p*_*pelvis*,*long*_ (m)
**Extension**	Torso falling	*p*_*torso*,*long*_ < *p*_*pelvis*,*long*_ (m)
		*p*_*torso*,*long*_ > *p*_*tpes*,*long*_ (m)
	Feet movement, hopping	*v*_*talus*,*linear*,*long*_ > 0.1 m/s
		*v*_*toes*,*linear*,*long*_ > 0.15 m/s
	Hip extension	*Θ*_*hip*_ < -11°
	Pelvis backwards movement	*v*_*pelvis*,*linear*,*ml*_ < = 0 m/s
**Stabilization**	Torso falling	*p*_*torso*,*long*_ < *p*_*pelvis*,*long*_ (m)
	Feet movement	*v*_*talus*,*linear*,*long*_ > 0.1 m/s
		*v*_*toes*,*linear*,*long*_ > 0.3 m/s
	Bad posture	*P*_*pelvis*,*ml*_ < *p*_*talus*,*ml*_ (m)
		*p*_*torso*,*ml*_ > *p*_*toes*,*ml*_ (m)
		-8° > *Θ*_*hip*_ > 8°

^a^Mediolateral.

^b^Longitudinal.

**Table 4:** Phase-dependent penalizations in the cost function for the 2-phases controller optimization.

Phase	Condition^a^	Penalizations	Cost function
**Flexion momentum**	No conditional^b^	Suboptimal acceleration	*a*_*torso*,*linear*,*ml*_ < = 0.8 m/s^2^
**Merged phase**	*Θ*_*hip*_ > 44°	Torso and pelvis backwards movement	*v*_*torso*,*linear*,*ml*_ < = 0 m/s
			*v*_*pelvis*,*linear*,*ml*_ < = 0 m/s
	8*° < Θ*_*hip*_ < 77°	Hip flexion	*ω*_*hip*_ > = 10°/s
		Knee flexion	*ω*_*knee*_ > = 0°/s
	*Θ*_*hip*_ < = 8°	Hip angle range	-8° > *Θ*_*hip*_ > 8°
		Knee flexion	*Θ*_*knee*_ > = 11°
	No conditional	Torso falling	*p*_*torso*,*long*_ < *p*_*pelvis*,*long*_ (m)
		Knee overextension	*Θ*_*knee*_ < -15°
		Feet movement	*v*_*talus*,*linear*,*long*_ > 0.1 m/s
			*v*_*toes*,*linear*,*long*_ > 0.3 m/s

^a^Conditional statements under the penalizations on the right are applied.

^b^ No conditional means that the penalizations on the right are applied along the entire phase.

### Optimization

For each model, we ran ten parallel simulations using a 3.60 GHz Intel Xeon W-1370P processor. The maximum duration for each simulation was 20s. The optimization process was set to stop after 10000 iterations of using the Covariance Matrix Adaptation Evolutionary-Strategy (CMA-ES), implemented on the SCONE software [12], which is based upon OpenSim [11]. The simulations were run on this framework, where the controllers (Eqs 2–4) and the cost function were coded as custom Lua scripts. The parameters to optimize are the different gains from Eq 2, length offset, tonic stimulation, and position reference. In total, 641 parameters were optimized for the 4-phases controller, and 241 for the 2-phases controller.

The CMA-ES is a derivative-free optimization algorithm to solve problems with a multi-variate normal search distribution by minimizing a unimodal cost function [13]. This algorithm is widely used for neuromuscular simulations because the cost function is evaluated independently in each iteration, making it relatively resilient to the inherent noise of biomechanical systems, and because an increase of the size of the problem (the number of parameters) can be easily counteracted by increasing the generation size [19]. The algorithm creates variations of the initial parameters (*mutation*) that are selected according to their fitness (*natural selection*) in the cost function. The cycle is closed when the variations of the parameters are *recombined* to create the initial parameters of the next iteration. Besides *natural selection*, *mutation* and *recombination*, this algorithm can present *genetic drift*, that is the change in the frequency of the variations of a gene in a population due to random chance [20]. Applied to genetic algorithms, *genetic drift* causes some parameters to be continuously updated randomly with an undesirable convergence, without sufficient exploration in the search space [21]. Genetic drift can be present in the optimization process of the 4-phases and the 2-phases controllers, because phases are presented sequentially. The parameters of one phase need to be optimized to complete the subtask and start the next phase, until that moment, the parameters of posterior phases are randomly updated. When the non-random update starts, these parameters cause postures and movements that would be penalized by the constraints of the cost function. Thus, the optimization process may be forced to find suboptimal solutions in previous phases. The additional parameters controlling the transitions between phases in our simulation (see *Sit-to-stand Phases* section) have the purpose of starting the next phase in the case its kinematic characteristics appear prematurely.

### Data analysis

All data resulting from the simulation performed in SCONE was processed on MATLAB 2022a. Algorithms for detecting the kinematic phases of STS were applied to the results of the subjects and both controllers. These algorithms use the definitions for the end of each kinematic phase according to the standard kinematic description [3] (Table 1), with some small changes due to the characteristics of the biomechanical model we used (Table 5). Note that the kinematic phases emerge from the execution of the modelled phases implemented in the controllers and they do not necessarily match.

**Table 5:** Comparison between algorithms for detection of kinematic phases.

Kinematic phases	Experimental data	Controllers
**Flexion momentum**	Vertical force on the seat reaches the lower plateau	Vertical force on the seat reaches 0N
**Momentum transfer**	Maximum dorsiflexion	Minimum angle of the shank respect to the positive x axis
**Extension**	Hip extension velocity < 0.0065°/s	Hips extension acceleration < 0.0065°/s^2^
**Stabilization**	Not applied	Hips extension acceleration < 0.0015°/s^2^
**Standing**	Not applied	End of the simulation

We used the lower point of the first derivative of the vertical force applied on the seat to detect the completion of the *flexion momentum* for the subjects (buttocks lift-off). The force applied on the seat in the simulation were irregular (the musculoskeletal model rests on a sphere), we used the moment this force reaches 0N instead. The moment of maximum dorsiflexion of the ankle is used to indicate the end of the *momentum transfer* phase. As the musculoskeletal model often rises the feet around the first half of the simulated STS cycle, we used the minimum angle of the shank with respect to the positive *x* axis to signal this transition in the analysis of the simulated results. The *Extension* ends when the hip extension velocity reaches 0°/s for the first time. However, as subjects performed a stand-to-sit after the STS, that event did not occur in the cycle of all the subjects, and we decided to detect the end of the *extension* phase when the subjects lowered the hip extension velocity more than 0.0065°/s for first time. In the case of the controllers, hips are slightly extending after the biomechanics model reached a normal stance position. Thus, the end of the phase was detected when the acceleration of the extension in the hips decreased below 0.0065°/s^2^. Other authors [3, 4] decided not to include the *stabilization* phase in the analyses because the movements related with rising could not be distinguished from the normal sway of the joints when standing. However, we needed to detect the end of the *stabilization* phase to compare the duration of the stance position between controllers. The end of the stabilization phase is established when the acceleration of the hips lower below 0.0015°/s2_._ The detection of the *stabilization* phase was not applied to the experimental data. *Standing* lasts until the end of the simulation.

A simulation is considered successful when all the phases of the STS are achieved consecutively and the *Extension* phase lasts no more than 1 second, as [3] specified. Standing longer than 3 seconds is also a requisite for STS achievement, to avoid simulations that led to inherently unstable postures. Simulations with standing lasting less than that period were discarded.

In our analysis, the STS cycle (Fig 2) is extracted and length-normalized to 100% (100 data points), considering the beginning of the simulation as 0 and the end of the *Extension* phase as 100%. This choice is for allowing the comparison between experimental and simulated results. The cycles from the simulated data are then averaged and compared to the experimental STS cycle. We compared the experimental and synthesized values for the joint angles in the sagittal plane and the normalized muscle activations. The comparison is supported by a cross-correlation study with 95% of confidence interval, as Geyer & Herr [9] and Choi & Bastian [22] used. We accepted there is a cross-correlation when the cross-correlation coefficient ® is higher than 0.7. The lag of the simulated sequences respect to the experimental ones in the STS cycle is denoted by Δ.

## Results

Both the 2-phases and 4-phases models were able to achieve STS kinematics comparable with the experimental data. For the 4-phases controller 8 out of 10 optimizations converged successfully and 4 out of 8 simulations achieved a physiological STS. The 2-phases controller achieved 9 out of 10 converged optimizations and 8 out of 9 successful STS. In terms of STS cycle, the times of completion of each phase for the controllers are consistent with the experimental results (Table 6). However, for the 4-phases controller, the second phase is longer compared to the 2-phases controller and the experimental results, and consequently the third phase is shorter (Table 7). The 2-phases controller has also been shown to result in simulations where balance is maintained longer with respect to the 4-phases controller.

**Table 6:** Comparison of the three first phases between experimental, 4-phases and 2-phases model.

Phase	Experimental [%]	4-phases controller [%]	2-phases controller [%]
**Flexion momentum**	35 ± 5	30.25 ± 4.57	37.47 ± 2.49
**Momentum transfer**	51 ± 4	65.03 ± 2.24	56.02 ± 2.33
**Extension**	98 ± 1	98.27 ± 0.37	98.12 ± 0.22

STS cycle [%].

**Table 7:** Comparison between 4-phases controller and 2-phases controller along the STS simulation.

Phase	4-phases controller [s]	2-phases controller [s]
**Flexion momentum**	0.43 ± 0.01	0.51 ± 0.01
**Momentum transfer**	0.98 ± 0.46	0.79 ± 0.39
**Extension**	1.45 ± 0.2	1.36 ± 0.1
**Stabilization**	1.84 ± 0.19	1.82 ± 0.09
**Standing**	6.6 ± 0.38	12.45 ± 1.12
**End of simulation**	6.99 ± 0.2	13.29 ± 1.13

Time of simulation [s].

**Fig 4:**
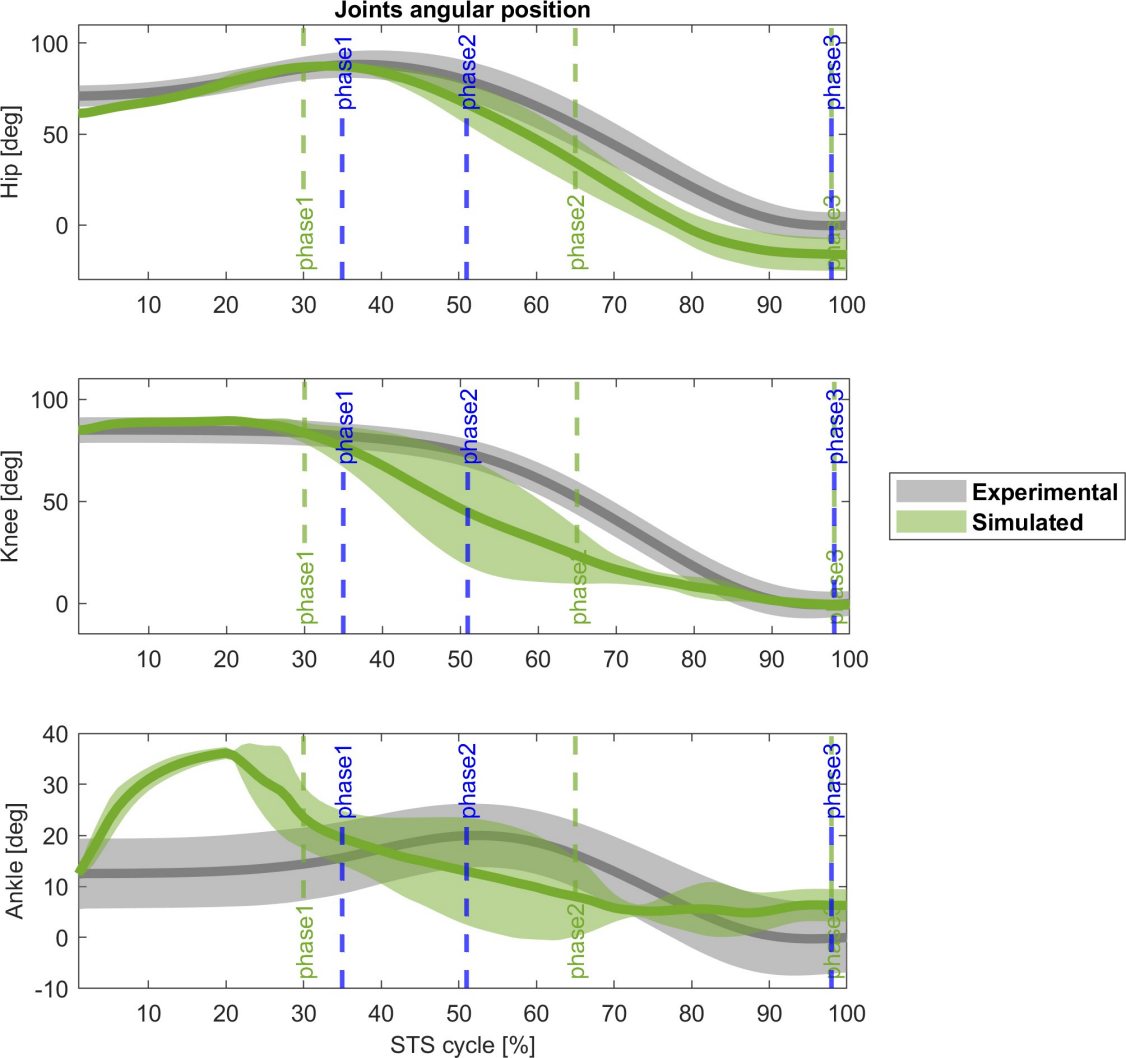
Experimental (grey) and 4-phases controller (green) joint angles [deg] comparison (mean ± std). Blue vertical lines: experimental cycle times; green vertical lines: 4-phases controller cycle times. Cross-correlation coefficient: r(hip) = 0.99 (Δ = 0%), and r(knee) = 0.93 (Δ = 0%), r(ankle) = 0.68 (Δ = -25%).

Fig 4 reveals the kinematics of the 4-phases controller matches the experimental data. Cross-correlation coefficients confirm the similarity for hip and knee angles between simulations and real data observed in the figures, (r_hip_ = 0.99, Δ = 0%; and r_knee_ = 0.93, Δ = 0%), while more marked differences for the ankle (r_ankle_ = 0.68, Δ = -25%).

At the 20% of the STS cycle, the hip and the knee extend sooner than the experimental subjects, with the hip finishing the cycle without correcting this deviation. The ankle has an abnormal dorsiflexion in the first phase, is more plantarflexed along the *momentum transfer* and the *extension* and finishes the cycle with an accentuated dorsiflexion again.

**Fig 5:**
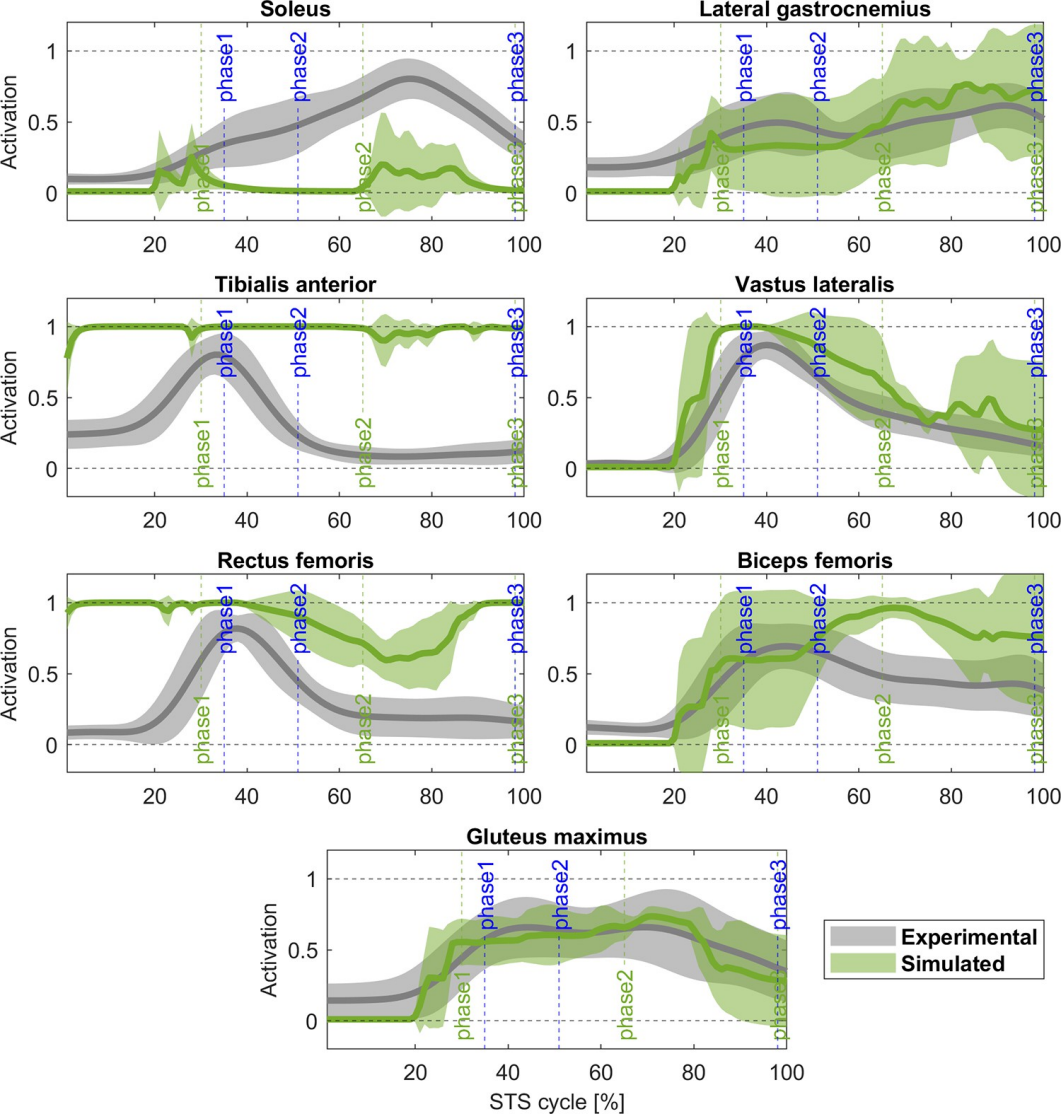
Experimental (grey) and 4-phases controller (green) muscle activation comparison (mean ± std). Activations is normalized between 0.1 (tonic activation) and 1. Blue vertical lines: experimental cycle times; green vertical lines: 4-phases controller cycle times. Cross-correlation coefficient: r(SOL) = 0.45 (Δ = 0%), r(GAS) = 0.93 (Δ = 0%), r(TA) = 0.31 (Δ = 12%), r(VAS) = 0.96 (Δ = 0%), r(RF) = 0.51 (Δ = 60%), r(BF) = 0.77 (Δ = 0%), r(GLU) = 0.96 (Δ = 0%).

**Fig 6:**
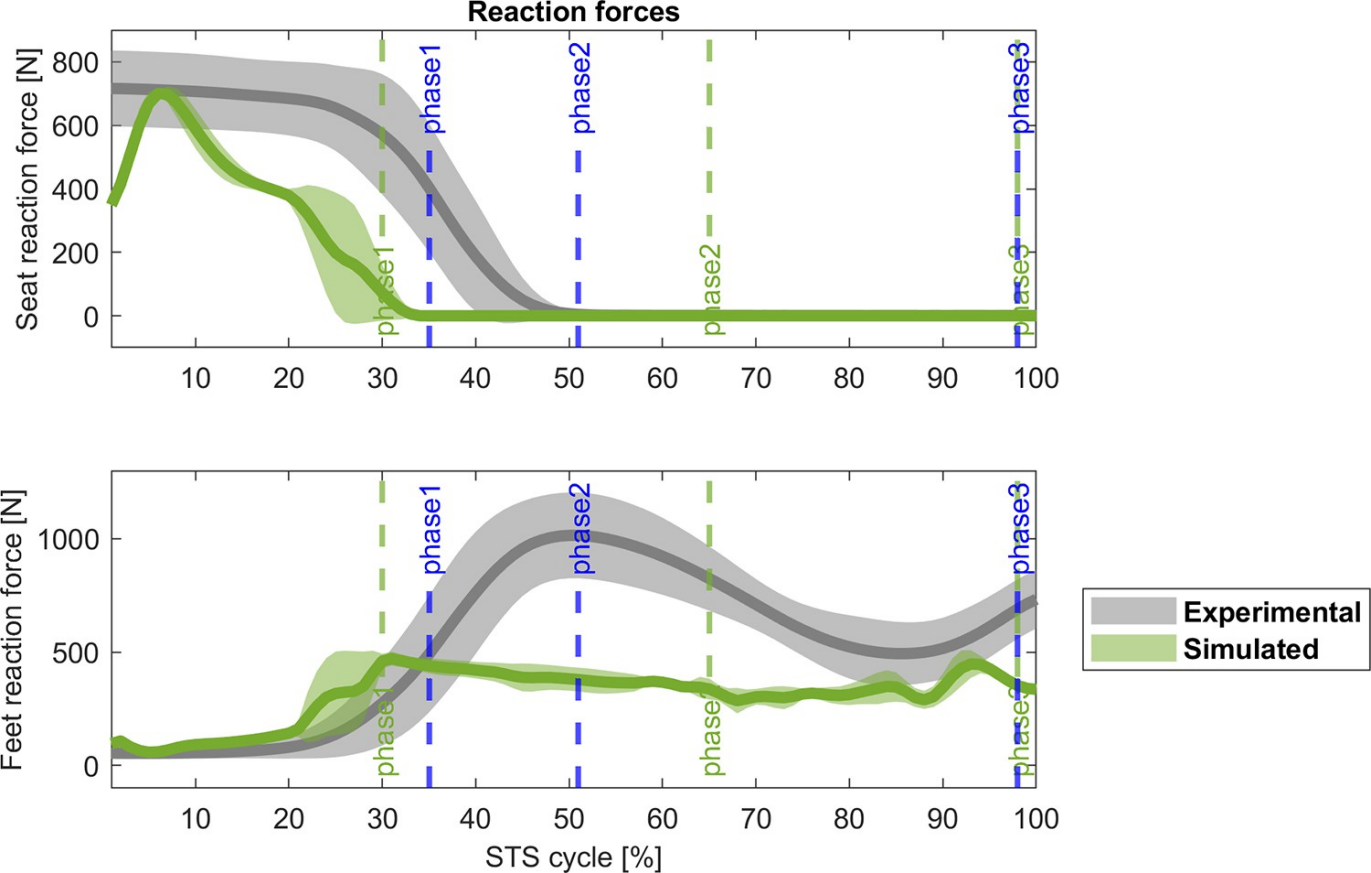
Comparison (mean ± std) of the reaction forces [N for the 4-phases controller. Grey: experimental data, green: simulated data; blue vertical lines: experimental cycle times; green vertical lines: 4-phases controller cycle times. r_Fseat_ = 0.86, Δ = -3%; r_Fground_ = 0.91, Δ = 0%.

The abnormal behaviour of the ankle is correlated with the activity of the muscles controlling this joint. In Fig 5, TA is remarkably overactivated (r_TA_ = 0.31, Δ = 12%) while the SOL has a lower activity compared with its experimental counterpart (r_SOL_ = 0.45, Δ = 0%). The other muscle with a low cross-correlation coefficient is the RF (r_RF_ = 0.51, Δ = 60%). The rest of the muscles cross-correlate with the experimental data (r_GAS_ = 0.93, Δ = 0%; r_VAS_ = 0.96, Δ = 0%; r_GLU_ = 0.96, Δ = 0%; r_BF_ = 0.77, Δ = 0%). Even though the reaction force on the seat and the ground (Fig 6) reveals a good correlation (rFy = 0.86, Δ = -3%; r_Fground_ = 0.91, Δ = 0%), the magnitude is noticeable different.

**Fig 7:**
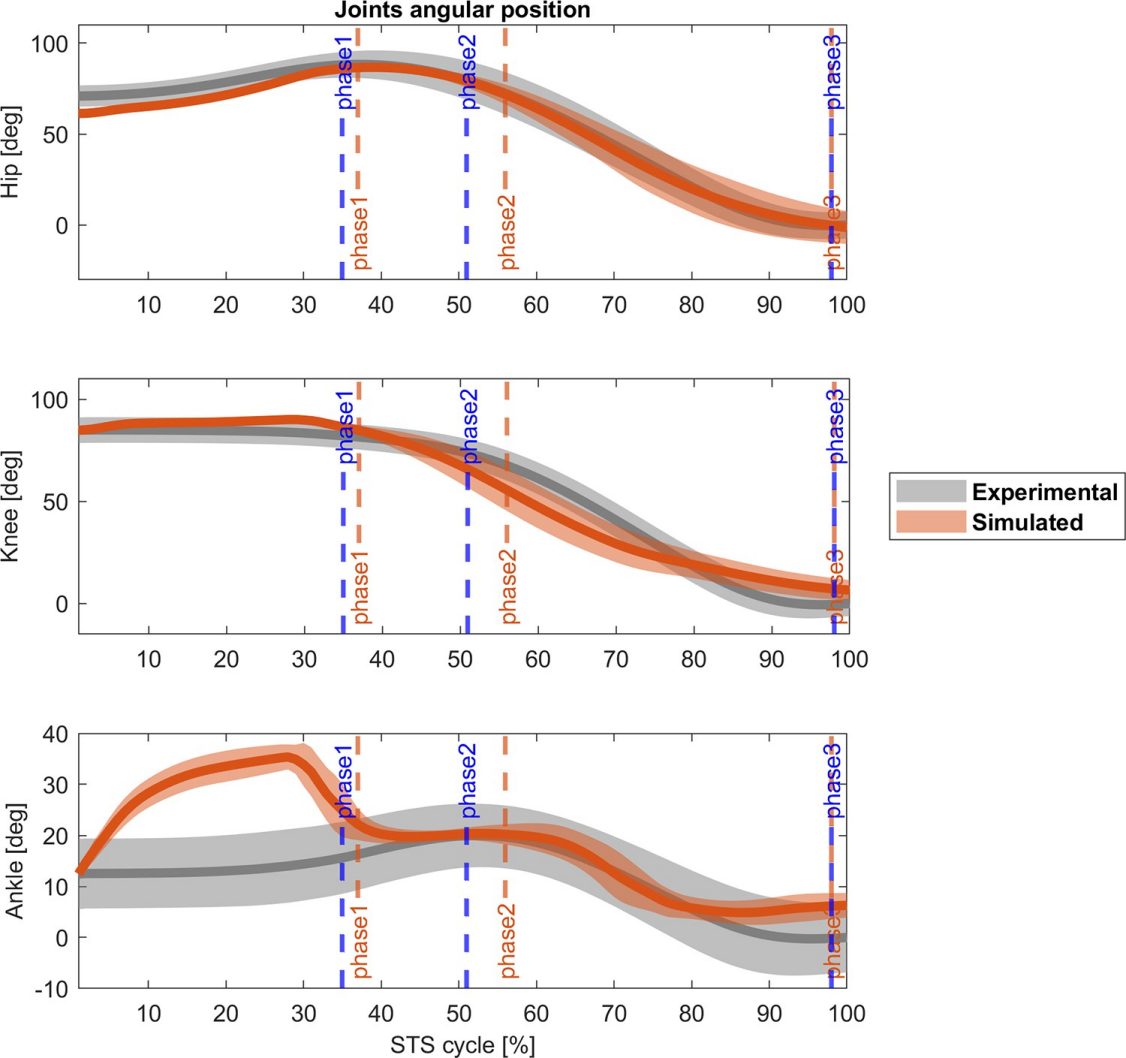
Experimental (grey) and 2-phases controller (orange) joint angles [deg] comparison (mean ± std). Blue vertical lines: experimental cycle times; orange vertical lines: 2-phases controller cycle times. Cross-correlation coefficient: r(hip) = 0.99 (Δ = 0%), and r(knee) = 0.97 (Δ = 0%), r(ankle) = 0.67 (Δ = -8%).

**Fig 8:**
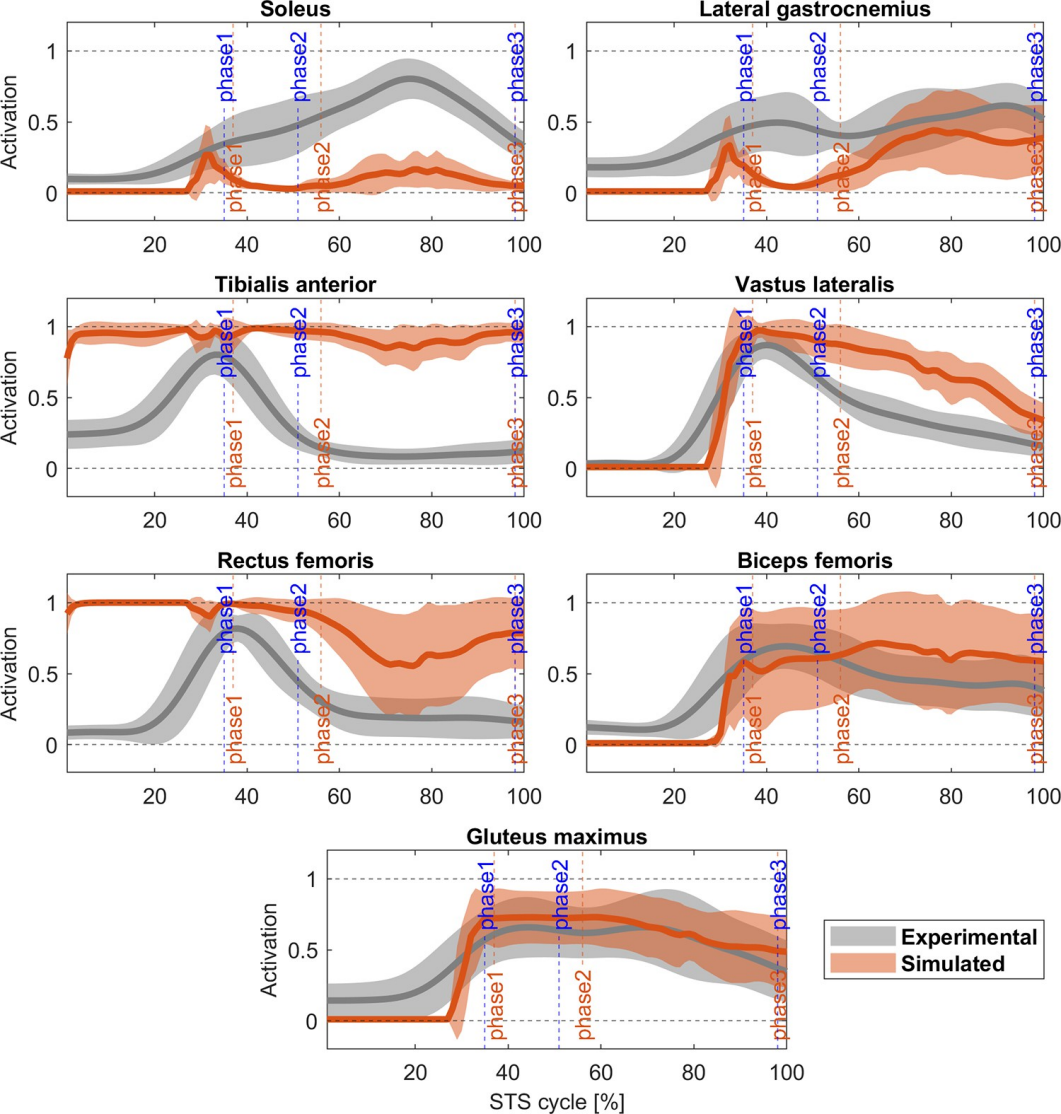
Experimental (grey) and 2-phases controller (orange) muscle activation comparison (mean ± std). Activations is normalized between 0.1 (tonic activation) and 1. Blue vertical lines: experimental cycle times; orange vertical lines: 2-phases controller cycle times. Cross-correlation coefficient: r(SOL) = 0.65 (Δ = 0%), r(GAS) = 0.79 (Δ = 0%), r(TA) = 0.58 (Δ = 11%), r(VAS) = 0.85 (Δ = 3%), r(RF) = 0.48 (Δ = -18%), r(BF) = 0.83 (Δ = 3%), r(GLU) = 0.96 (Δ = 0%).

**Fig 9:**
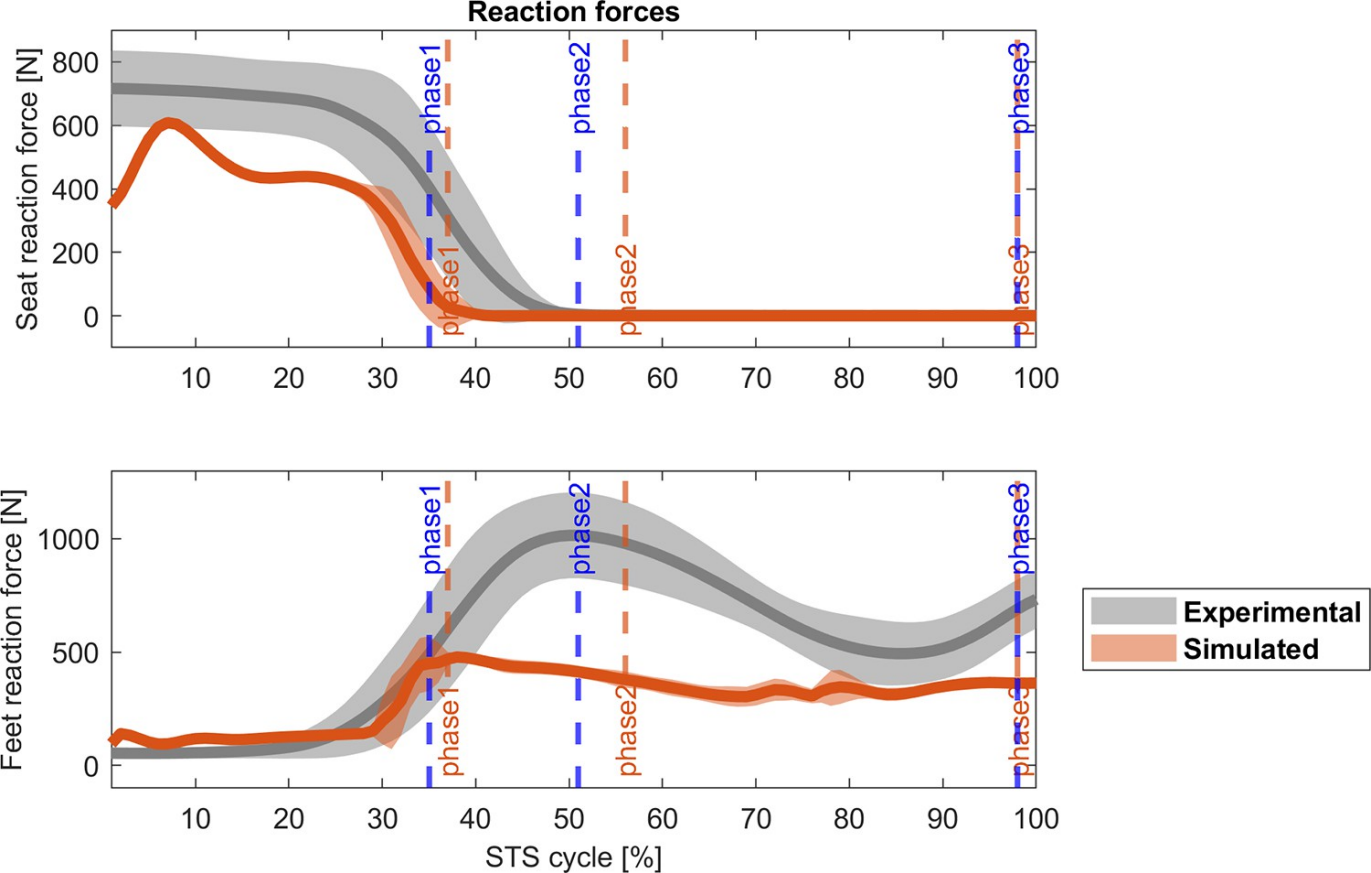
Comparison (mean ± std) of the reaction forces [N] for the 2-phases controller. Grey: experimental data, orange: simulated data; blue vertical lines: experimental cycle times; orange vertical lines: 2-phases controller cycle times. r_Fseat_ = 0.96, Δ% = 0; r_ground_ = 0.90, Δ% = 3.

Figs 7 and 8 present the results for the 2-phases controller. Joint angles have a similar behaviour compared to the 4-phases controller. The cross-correlations are positive for hips and knees (r_hip_ = 0.99, Δ = 0%; r_knee_ = 0.97, Δ = 0%), but negative for ankle (r_ankle_ = 0.67, Δ = -8%), again. The ankle angle also shows the persistent toe lifting until the 40% of the STS cycle but correlates better with the experimental ankle angular position for the rest of the cycle. Even though hips present a good correlation at the end of the cycle, knees and ankles are slightly flexed. Regarding the muscle activation, vastus lateralis, biceps femoris, gluteus maximus, and lateral gastrocnemius match the experimental data (r_VAS_ = 0.85, Δ = 3%; Δ = 0%; r_BF_ = 0.83, Δ = 3%; r_GLU_ = 0.96, Δ = 0%; r_GAS_ = 0.79, Δ = 0%). However, soleus (r_SOL_ = 0.65, Δ = 0%), tibialis anterior (r_TA_ = 0.58, Δ = 11%), and rectus femoris (r_RF_ = 0.48, Δ = -18%) present the same problem as in the 4-phases model and their activity is generally lower or higher than the average experimental activation. Fig 9 shows the correlation between experimental and simulated reaction force on the seat (r_Fseat_ = 0.96, Δ = 0%), and the ground (r_Fground_ = 0.90, Δ% = 3). Both values are lower than those observed in subjects, again. Overall, the STS biomechanics, the timing of the kinematic phases and the duration of the standing are better for the 2-phases controller than the 4-phases controller. Also, while just four out of ten of the optimizations of the latter achieved a physiological STS, the optimizations for the 2-phases controller achieved eight out of ten successful STS.

To summarize, the 2-phases controller presented phase timing more aligned with experimental data, simulations resulting in more stable final postures and kinematics closer to experimental data with respect to the 4-phases controller. The optimization of the 2-phases controller was also simpler and more efficient, being the number of successful STS twice in the 2-phases controller than in the 4-phases controller.

## Discussion

Here we developed two neural controllers for predictive simulations of STS based on vestibular and muscle-length reflexes. The first controller is based on a parametrization of the reflexes following a kinematic description of the phases of STS, while the second proposes a simplified solution that groups the reflex characteristics of the *momentum transfer*, *extension*, and *stabilization* tasks.

Both controllers can achieve functional STS movements as defined by previous literature [3, 4]. The results obtained on the sagittal joint angles generally match the data collected from the healthy study participants. The exceptions were the TA and the RF, overactivated compared to their experimental counterpart. The activity of the SOL is also lower than expected. All of this causes the abnormal angle in the ankle joint. This discrepancy with respect to the experimental data can be easily accounted by the configuration of the musculoskeletal model. Originally based on a gait biomechanical model, our model does not present a lumbar joint between the torso and the pelvis, nor thoracic muscles to flex it. The rectus abdominis plays a major role during STS, especially during the *flexion momentum* [23]. The lack of these two elements requires finding solutions in other flexors of the hip which could generate the proper forward flexion of the torso. The psoas and iliacus were discarded because including these two muscles caused the simulations for both controllers to not converge. However, the exclusion of these muscles it is not expected to affect results significantly, as their contribution during the STS cycle has been reported to be lower than 200 N [4], and, when these muscles are weakened, changes in the kinematics are negligible [24]. The only actuator in the model able to accomplish the *flexion momentum* phase was the rectus femoris. Bobbert et al. also concluded that rectus femoris had a major role in the generation flexion moment at the hip [25]. However, in our simulations, the rectus femoris was forced to act out of its range of hip flexion (0-45°) [26], causing overactivation. The rectus femoris is also a biarticular joint and during the *flexion momentum* the knees were prone to extend and slide the feet forward. The tibialis anterior counteracts the feet moving forward and the BoS moving away from the CoM. Tibialis anterior has an important role in the *flexion momentum* preparing the ankle for the *momentum transfer* [27, 28]. The additional task of counteracting feet movements seems to cause the disproportional activation of the tibialis anterior, and we cannot discard the possibility that this overactivation could interfere with TA’s original function and the stabilization of the ankle in the following phases. The dorsiflexion of the ankle during the first phase is likely a result of this overactivation. The discrepancies related to the reaction forces in both controllers can be attributable to the fact that our model rests with a ball of reduced size (4 cm of radius) on the seat, instead of the buttocks that a human body uses.

We cannot exclude the presence of genetic drift during the optimization process. While some authors [29] consider that the impact of the genetic drift depends on the application, in our case, where the controller is made of sequential phases, phase-specific sets of parameters were updated randomly, worsening the exploratory behaviour in the search space. As the random update is prolonged as the number of phases increase, the 4-phases controller was likely affected by this effect more than the 2-phases controller. Hence, while eight out of ten optimizations converged to a physiological STS for the 2-phases controller, six optimizations of the 4-phases controller had to be discarded. The number of iterations needed to optimize the first three phases was too large in most of the cases. At the time that the *stabilization* phase was reached, the randomly updated parameters caused sudden and desynchronized movements, forcing the optimization to find suboptimal solutions on previous phases. The 2-phases controller did not have that problem as the *flexion momentum* was quickly optimized, and the parameters of the *merged phase* underwent genetic drift just during a small number of iterations.

Even with the limitations due to the simplicity of the biomechanical model and the likely presence of genetic drift, the results of our simulations allow to infer some new insights about the nature of the underlying control of STS. We saw that the STS controller can be encoded in phases, but the success of the 2-phases simulations suggests that a simple reflex-based architecture could suffice for explaining the pattern of movement observed in experimental STS.

The constraints and simplicity of the model and of the implementation of the covariance matrix adaptation evolutionary strategy (CMAES) algorithm do not allow to assume that such reflex configurations represent the true nature of the STS. But the idea of one set of reflexes controlling different dynamics is interesting and deserves further explorations. If the emerged properties of a unique set of reflexes working together can allow the control of different motor dynamics at the same time, it is possible that the neural systems may employ, at least partially, this kind of organization.

Bobbert et al. [25] or Caruthers et al. [4] created biomechanical models to study the kinetics and muscle dynamics with no underlying assumption about the organization of the neural activity. An et al. [30], on the other hand, proposed a neuromuscular model where the STS is the product of four synergies coded at the spinal cord. The synergies were determined by spatial coefficients that codified the activation of a muscle in a synergy, and by temporal coefficients that activate a synergy in a specific moment of the STS cycle. This model has been able to explain standing up motion and, in successive implementations, being responsive to sensorial inputs [31] or classify STS performance according to the temporal features of these synergies [32]. The success of this model inspires the possibility of developing a reflex-based controller for STS implemented on the synergies rather than the single muscles. A model where a reduced number of sensorial inputs can trigger and modulate the activity of a group of muscles (e.g., synergistic polysynaptic reflexes) to achieve a specific subtask would simplify the number of parameters and interactions presented in this work. As the synergies can work simultaneously, the genetic drift can also be reduced.

The plausibility of neuromechanical predictive models depends on their capacity to reproduce characteristics of motions observed in experimental tasks. Nevertheless, this plausibility does not ascertain that the principles on which a model is based reflect the actual neurophysiology. Our simulations are successful in accomplishing STS, while bearing some differences in the neuromechanical characteristics of the task execution, that are mostly related to the simplicity of the biomechanical model that we used. The soleus showed discrepancies with respect to the experimental results, especially when considering the role of these muscles in the STS tasks. Study participants show activations in this muscle much higher than those observed in the simulation, even though they are time-correlated. In the *momentum transfer* phase, legs create the new BoS and start bearing the weight of the body. That means that force feedback of the extensors of lower limbs could be proposed to drive this phase similarly as it happens during gait [6, 33, 34]. Including this type of reflexes could solve the gap between experimental and simulated results for the soleus.

Both controllers, 4-phases, and 2-phases, need the torso to create enough momentum to successfully raise the body. The values of the minimum forward acceleration of the torso and the angular position of the hips at lift-off are thus essential during the optimization process. Hughes et al. [35] mentioned these two values define three strategies for chair rising. In a momentum transfer strategy, torso momentum is maximized and would be preferred by young and healthy elderly subjects. Impaired elderly individuals prefer maximizing the angular position of the hips in a stabilization strategy, being the hybrid strategy a combination of the other two. Our models use the momentum transfer strategy. It needs to be noted that the reflex controllers herein presented are not able to mimic the stabilization strategy because it would require to add degrees of freedom and the rectus abdominis in the torso to control forward flexion. A controller able to switch strategies is necessary for extrapolating results of neuromuscular models representing aged population, where subjects tend to change to the stabilization strategy.

We forced our musculoskeletal model to be symmetrical for the muscular activity, because we do not have access of the muscular data for both sides and to reduce the number of parameters to be optimized regarding computational efficiency. However, Caruthers et al. [4] pointed that STS is asymmetrical even though both sides move synchronously. Although it is likely that lateral dominance would influence STS, to mimic such a behaviour in a simulation, this would require to be explicitly coded in the controller.

The duration of the different phases of STS, expressed as percentages of the whole task duration, are similar between experimental and simulated results, however, in our models, the hips were still extending during the *stabilization* phase. Yoshida et al. [18] supports that time and intensity of the pattern of the muscle activations can be altered by sensorial disruption. They indicated that visual input could have an important role in the *stabilization* phase. In our model where postural control relies on vestibular information the visual inputs are not modelled, thus the abnormal fourth phase (*stabilization*) that can be observed in our simulation could be due to the lack of visual input. However, this observation cannot be confirmed in our study as to confirm it is necessary to compare our simulations with data recorded during STS movements performed while blindfolded.

In conclusion, we show that simple reflex controllers can explain physiologically normal STS and stable standing, and the results of the 2-phases controller suggests that reflex characteristics are maintained during most of the STS task. If we consider rising and standing up motions as two separate motor behaviours, the 2-phases controller offers a simplistic model of a control architecture. Our results show that the neural control of STS can be modelled as a simple set of reflexes where phases do not need to be coded. This controller must include vestibular feedback as the main navigation system, and muscle force feedback and muscle length feedback for postural control. Although this controller needs to be validated on a more complex biomechanical model, the initial results herein presented suggest that such controller can represent a framework to investigate how healthy individuals may alter the control of STS in the presence of assistive or resistive forces, such as those provided by an exoskeleton.
